# Brain pathology in myotonic dystrophy: when tauopathy meets spliceopathy and RNAopathy

**DOI:** 10.3389/fnmol.2013.00057

**Published:** 2014-01-09

**Authors:** Marie-Laure Caillet-Boudin, Francisco-Jose Fernandez-Gomez, Hélène Tran, Claire-Marie Dhaenens, Luc Buee, Nicolas Sergeant

**Affiliations:** ^1^Alzheimer and Tauopathies, Faculty of Medicine, Jean-Pierre Aubert Research Centre, Institute of Predictive Medicine and Therapeutic Research, Inserm, UMR 837Lille, France; ^2^University of Lille Nord de France, UDSLLille, France

**Keywords:** RNAopathy, tauopathy, splicing, myotonic dystrophy, Alzheimer's disease

## Abstract

Myotonic dystrophy (DM) of type 1 and 2 (DM1 and DM2) are inherited autosomal dominant diseases caused by dynamic and unstable expanded microsatellite sequences (CTG and CCTG, respectively) in the non-coding regions of the genes *DMPK* and *ZNF9*, respectively. These mutations result in the intranuclear accumulation of mutated transcripts and the mis-splicing of numerous transcripts. This so-called RNA gain of toxic function is the main feature of an emerging group of pathologies known as RNAopathies. Interestingly, in addition to these RNA inclusions, called foci, the presence of neurofibrillary tangles (NFT) in patient brains also distinguishes DM as a tauopathy. Tauopathies are a group of nearly 30 neurodegenerative diseases that are characterized by intraneuronal protein aggregates of the microtubule-associated protein Tau (MAPT) in patient brains. Furthermore, a number of neurodegenerative diseases involve the dysregulation of splicing regulating factors and have been characterized as spliceopathies. Thus, myotonic dystrophies are pathologies resulting from the interplay among RNAopathy, spliceopathy, and tauopathy. This review will describe how these processes contribute to neurodegeneration. We will first focus on the tauopathy associated with DM1, including clinical symptoms, brain histology, and molecular mechanisms. We will also discuss the features of DM1 that are shared by other tauopathies and, consequently, might participate in the development of a tauopathy. Moreover, we will discuss the determinants common to both RNAopathies and spliceopathies that could interfere with tau-related neurodegeneration.

## Introduction

Myotonic dystrophy (DM) of type I (DM1), which is also referred to as dystrophia myotonica or Steinert's disease, is the most common form of adult-onset muscular dystrophy and affects at least 1 in 8000 people worldwide. DM1 is an inherited autosomal dominant disease caused by the dynamic and unstable expansion of a trinucleotide CTG repeat motif in the 3′ UTR of the *DMPK* gene located at q13.3 on chromosome 19 (Brook et al., 1992). Affected individuals carry CTG copy numbers greater than 50 and present a highly variable phenotype, ranging from asymptomatic to a severe congenital form of the disease. The length of CTG expansion has been associated with the severity of the pathology and is dependent on both meiotic and somatic instability (Mahadevan et al., 1992; Harley et al., 1993; Wong et al., 1995; Martorell et al., 1998). This inherited neuromuscular disease affects multiple organs, including skeletal and smooth muscles (distal muscular atrophy, myotonia, muscle weakness grip and wasting, percussion myotonia hatchet face, and ptosis), the heart (arrhythmia and conduction defects), the endocrine system (hyperinsulinemia), eyes (cataracts), gonads (atrophy), the central nervous system (CNS) (executive and visuoconstructive difficulties, facial emotion recognition impairment, and neuropsychiatric symptoms), and the peripheral nervous system (axonal peripheral neuropathy) (cf reviews: Schara and Schoser, 2006; Turner and Hilton-Jones, 2010; Romeo, 2012).

The mechanisms underlying DM1 physiopathology have recently begun to be elucidated. DM1 is the first identified disease resulting from a repeat expansion in a non-coding region of mRNA. Thus, the toxic effect of the CTG expansion seems to be more associated with the expression of mutated DMPK RNA than with changes in the expression of the DMPK protein. Substantial evidence supports a pathogenic role for these non-coding repeats at the RNA level, such as the discovery of a second type of DM, DM2 (or PROMM, proximal myotonic myopathy), which is due to a CCTG repeat expansion in another non-coding part of another transcript, the first intron of the *ZNF9* gene (Liquori et al., 2001). The nuclear export of the mutated RNAs is defective, and they subsequently accumulate and aggregate to form so-called nuclear foci, which then recruit and sequester RNA-binding proteins (RBP) [reviewed in Day and Ranum (2005), Schoser and Timchenko (2010)]. The identification of new pathologies presenting a similar RNA toxicity associated with repeat expansions, foci appearance, and the sequestration of RBP led to the identification of these disorders as RNAopathies (Renoux and Todd, 2012). The subsequent loss of function of these RBP leads to a disruption in RNA metabolism, including modifications of the alternative splicing of numerous pre-messenger RNAs in several tissues. This altered process highly contributes to the multisystemic effect of the DM1 mutation [reviewed in Kuyumcu-Martinez and Cooper (2006); Ranum and Cooper (2006)]. Other mechanisms might interfere with pathology development. The haploinsufficiency of the protein encoded by the mutated allele and the *cis*-effect of the mutation on neighboring genes have been explored (Fu et al., 1993; Novelli et al., 1993; Otten and Tapscott, 1995; Thornton et al., 1997). More recently, the mutation in DM1 has been suggested to have additional effects on the translation and stability of proteins, generation of toxic anti-sense transcripts, and expression of toxic homopolymeric peptide species through a non-ATG initiated translation pathway [recently reviewed in Klein et al. (2011), Sicot et al. (2011)].

Interest in the neurological aspects of DM has increased in the last several years. Reviews and workshops of these studies have primarily focused on clinical symptoms and neuroimaging (Axford and Pearson, 2013). This review highlights a particular aspect of DM neurological disorders, the tauopathy. Tauopathies are neurodegenerative diseases characterized by the intraneuronal aggregation of microtubule-binding Tau proteins. The aim of this review is to describe the different aspects of the Tau pathology observed in DM1, including clinical symptoms, brain histology, and molecular mechanisms. We will also discuss the features of DM1 that are shared by other tauopathies and neurodegenerative diseases and that might contribute to the development of tauopathies. Moreover, we will focus on the possible interactions between the mechanisms of RNAopathy, spliceopathy, and tauopathy in the development of DM brain pathology.

## DM neuropathological signs and tauopathy features

### Clinical symptoms

Cognitive impairment in DM1 has been clearly established. DM1 patients exhibit changes in personality traits and/or mood disorders (Rubinsztein et al., 1998; Meola et al., 2003; Antonini et al., 2006; Winblad et al., 2006b). The cerebral involvement of DM1 patients has been associated with difficulties in executive functions (D'Angelo and Bresolin, 2003), visuospatial/constructive abilities (Malloy et al., 1990), memory (Rubinsztein et al., 1997), facial emotion recognition (Winblad et al., 2006a), and psychomotor delay. Apathy (Rubinsztein et al., 1998), avoidance (Meola et al., 2003), depression and anxiety (Antonini et al., 2006), anhedonia, and decreased emotional participation are often the main neurological and clinical symptoms of this pathology (Bungener et al., 1998). Approximately one third of DM1 patients also suffer from excessive daytime sleepiness (EDS) that most likely results from CNS disturbance [reviewed in Laberge et al. (2013)]. Consistent with this observation, MBNL2 KO transgenic mice, which are characterized by neurodegenerative symptoms in the absence of obvious muscle alterations, develop hypersomnia (Charizanis et al., 2012). In the most severe cases of DM1 (congenital/juvenile form), mental retardation has also been described [reviewed in Machuca-Tzili et al. (2005); Schara and Schoser (2006)]. Furthermore, approximately half of these young patients also have autism spectrum disorders, the frequency of which is related to the number of CTG repeat (Ekstrom et al., 2008).

Brain involvement in DM2 is more controversial. Similar cognitive and behavioral dysfunctions are described, with milder manifestations compared with DM1 (Meola et al., 2003; Weber et al., 2010). In contrast to DM1, DM2 has not been associated with developmental abnormalities and thus does not cause severe childhood symptoms. This difference likely explains why no mental retardation similar to that reported in congenital and juvenile forms of DM1 has been described in DM2 patients.

### Neuroimaging

MRI studies have revealed global cerebral atrophy with dilated ventricles in DM1 (Censori et al., 1994; Antonini et al., 2004). No correlation has been observed between brain tissue volumes and the grade of pathology, disease duration, or CTG expansion. However, the potential correlation between brain tissue volume and CTG expansion remains unclear because these studies were performed using repeat lengths measured from blood cells and not from brain tissue, where the somatic instability of CTG expansion is particularly obvious and varies both with a single brain area and between different areas. Indeed, the expansion length can vary from 150 to more than 3000 CTG repeats (Sergeant et al., 2001; Dhaenens et al., 2011). Furthermore, a large difference in the CTG expansion length between muscle and blood has recently been reported, confirming that the CTG length depends on the tissue analyzed (Nakamori et al., 2013). Thus, the potential correlation between brain imaging alterations and CTG repeat length in the altered regions remains unclear. Future follow-up clinical studies are needed to support a correlation.

In DM1, cortical gray matter loss is primarily observed in the frontal, parietal, and occipital regions and in the superior and middle temporal gyrus, whereas subcortical gray matter loss is detected in thalamic and basal ganglia structures (Antonini et al., 2004; Weber et al., 2010; Minnerop et al., 2011). These areas are involved in cognitive dysfunctions and personality disorders, such as apathy, depression, anxiety, and deficits in attention, memory, and visuospatial function (Antonini et al., 2004). Cortical atrophy has also been observed in DM2, although to a lesser extent than in DM1 (Ota et al., 2006; Minnerop et al., 2008, 2011).

White matter lesions are more pronounced than gray matter lesions in the DM1 brain. Regression analyses have revealed associations between affected white matter and several clinical parameters in both DM1 and DM2, but no associations with neuropsychological performance have been described (Minnerop et al., 2011). A recent study of children and adolescents suggested a relationship between white matter damage and working memory (Wozniak et al., 2013). White matter lesions are detected throughout the whole brain in DM types 1 and 2, affecting association fibers, commissural fibers (primarily in the corpus callosum), and projection fibers in the brainstem and the internal and external capsules, which connect the prefrontal and temporal cortical areas with the striatum. Although white matter lesions are also present in the frontal regions of patient brains, lesions located within anterior temporal lobes are considered a characteristic feature in DM patients (Hund et al., 1997; Di Costanzo et al., 2001; Naka et al., 2002; Kassubek et al., 2003; Kornblum et al., 2004; Fukuda et al., 2005; Vielhaber et al., 2006; Minnerop et al., 2011). These lesions demonstrate familial aggregation in DM1 and could be progressive along the disease evolution in association with CTG length (Di Costanzo et al., 2001; Ota et al., 2006; Romeo et al., 2010; Minnerop et al., 2011). Moreover, “état criblé” in the cerebral deep white matter has recently been reported (Itoh et al., 2010). Furthermore, hypoperfusion and glucose hypometabolism in the frontal and temporal lobes have been observed in DM, although these features are present to a greater extent in the DM1 brain than in the DM2 brain (Meola et al., 1999; Weber et al., 2010).

### Histology

#### Foci

Mutated DMPK transcripts with abnormally expanded CUG repeats are retained and accumulated in RNA nuclear inclusions called foci, which were first observed in DM1 muscle biopsies (Taneja et al., 1995). These foci have also been observed in human DM1 brains, particularly in the neuronal cells of the cerebral cortex, hippocampus, dentate gyrus, thalamus, substantia nigra, and brain stem tegmentum. A weak detection of foci has also been reported in the oligodendrocytes of the subcortical white matter and corpus callosum (Jiang et al., 2004). These nuclear RNA inclusions sequester RNA-binding proteins, such as the splicing factors Muscleblind-like 1 (MBNL1) and MBNL2 (Miller et al., 2000; Fardaei et al., 2001, 2002; Jiang et al., 2004) and to a lesser extent, heterogeneous nuclear ribonucleoproteins (hnRNPs) H and F (Jiang et al., 2004; Kim et al., 2005; Paul et al., 2011). While hnRNP H has been suggested to prevent the nuclear export of the mutated transcripts, MBNL1 has been directly implicated in the formation and stabilization of foci [(Kim et al., 2005; Querido et al., 2011); detailed below]. Interestingly, focus formation has also been observed in the brains of transgenic DMSXL mice bearing more than 1000 CTG repeats (Huguet et al., 2012) and in neuronal progeny derived from human embryonic stem cells carrying the DM1 mutation, particularly neuronal cells with a motor neuron phenotype (Marteyn et al., 2011).

#### Neurofibrillary degeneration (NFD)

NFD is an age-related process that occurs during normal aging and is abnormally enhanced in neurodegenerative diseases referred to as “tauopathies”. NFD is characterized by the accumulation of intraneuronal argyrophilic fibers. This insoluble material corresponds to the accumulation and aggregation of hyperphosphorylated microtubule–associated Tau proteins, which form neurofibrillary tangles (NFTs) [reviewed in Buee et al. (2010)], mainly in neuron soma but also in neuropile threads, similarly to that observed in an AD brain, whereas Tau proteins of a healthy neuron are mainly located in the axon. First identified in the brains of Alzheimer's disease (AD) patients, NFD is now considered a common neuronal feature in nearly 30 tauopathies [reviewed in Sergeant et al. (2008)]. The spatiotemporal progression of the neurofibrillary lesions through the brains of AD patients can be subdivided in 6 or 10 stages according to the anatomopathological description of Braak and Braak (1991) or the biochemical analysis of more than 20 brain regions from 200 individuals, respectively (Delacourte et al., 1999). The density and topographic progression of NFTs have been correlated with disease severity and cognitive decline in AD patients (Duyckaerts et al., 1997; Berg et al., 1998; Giannakopoulos et al., 2003; Bennett et al., 2004). This observation strongly suggests a central role for fibrillar Tau inclusions in the pathophysiology and clinical symptoms of AD. Furthermore, the propagation of Tau pathology has recently been reported in animals after the injection of insoluble material obtained from animal models of AD, the brains of AD patients, or a lentivirus encoding wild type Tau (Clavaguera et al., 2009, 2013; De Calignon et al., 2012; Lasagna-Reeves et al., 2012; Caillierez et al., 2013).

This relationship between Tau inclusion and pathophysiology is supported by the identification of autosomal dominant mutations in the *MAPT* gene in various other tauopathies, such as fronto-temporal dementia (FTD); these mutations are sufficient to induce both clinical symptoms and Tau pathology [reviewed in Schraen-Maschke et al. (2008), Umeda et al. (2013)].

**Figure 1:**
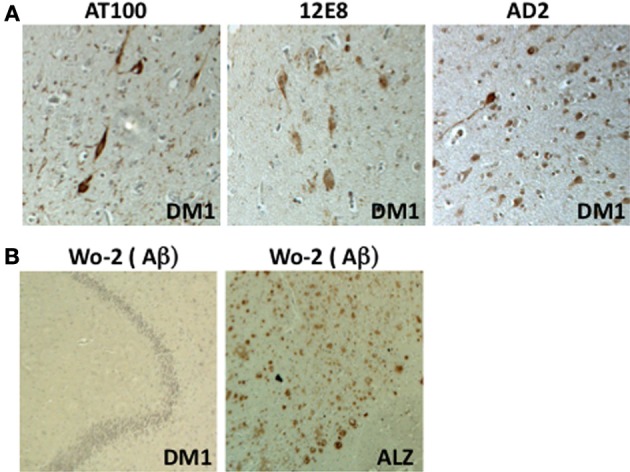
**Immunohistochemical analysis of the DM1 hippocampus (CA1 region). (A)** Detection of NFTs using three antibodies directed against Tau phosphorylated Ser202-205 (AT8), phosphorylated Ser 262 (12E8), and phosphorylated Ser396-404 (AD2) (x200). **(B)** Absence of amyloid detection using the Wo-2 antibody in samples obtained from individuals with DM1 (x100). Positive control: AD brain (Alz) (×100).

Is the cognitive dysfunction reported in DM1 associated with the development of a tauopathy? Indeed, NFTs have been observed in both DM1 and DM2 brains in the amygdala, CA1, hippocampus, entorhinal cortex, and temporal cortex, then with a topographic distribution similar to that reported for moderate Alzheimer disease although the topographic progression of Tau pathology during DM1 has not yet been clearly established (Figure 1) (Yoshimura et al., 1990; Vermersch et al., 1996; Delacourte et al., 1999; Maurage et al., 2005). Thus, DM can be considered as a tauopathy-associated disease solely based on the presence of NFTs (Yoshimura et al., 1990; Vermersch et al., 1996; Sergeant et al., 2001; Maurage et al., 2005; Oyamada et al., 2006; Itoh et al., 2010). Although a lower distribution of Tau inclusions throughout the brain is observed in DM patients compared with other pathologies, such as AD, the topographic distribution and expression of NFTs are still higher than that in unaffected individuals of the same age (Vermersch et al., 1996). Tau pathology progression in the DM brain is therefore considered moderate and is close to Braak and Braak stage 3–4 based on the number of NFTs observed.

Although all tauopathies are characterized by intraneuronal Tau aggregates, their clinical symptoms and histopathological criteria differ, including the structure of the aggregates (paired helical filaments, straight filaments or Pick bodies), the cerebral localization of degenerating neurons, the presence or absence of glial Tau inclusions (astrocytic plaques, tuft-shaped astrocytes, and oligodendroglial coiled bodies), and the association with other types of neuropathological lesions, such as extracellular amyloid deposits or Lewy bodies (LBs) [reviewed in Sergeant et al. (2005)]. In DM1 brains, no amyloid/senile plaques have been described (Figure 1), and α-synuclein inclusions (LBs) have scarcely been observed (Kiuchi et al., 1991; James et al., 2012) (Maurage and Sergeant, unpublished results). DM is therefore different from AD or dementia with Lewy bodies (DLB) (Kiuchi et al., 1991). However, as in many age-related neurodegenerative disorders, a strong gliosis has been observed (Yoshimura et al., 1990; Ono et al., 1995). Although neurodegenerating neurons typically contain NFTs, the ultrastructure of these DM Tau aggregates remains unknown.

**Figure 2:**
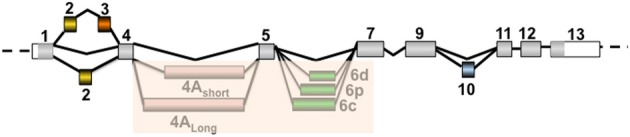
**Different splicing patterns of Tau- alternative exons leading to the main expression of six isoforms in the adult brain: 2N3R, 1N3R, 0N3R, 2N4R, 1N4R, and 0N4R**. The isoforms 0N, 1N, and 2N correspond to the exclusion or inclusion of only exon 2 or both exons 2 and 3. Note that exon 3 insertion is dependent on exon 2 insertion. The isoforms 3R and 4R correspond to the exclusion or inclusion of exon 10, respectively. Note that this figure only indicates the alternative splicing of coding exons. The coding regions are colored in gray for constitutive exons and in yellow, orange, pale purple, green, and blue for alternative exons 2, 3, 4A, 6, and 10, respectively. The inclusion of exons 4A and 6 occurs rarely in the brain and is more prominently detected in the peripheral nervous system, and the insertion of these exons varies according to the 3′ splicing site used. These two exons are shaded because the corresponding protein isoforms are not detected in the brain.

**Figure 3:**
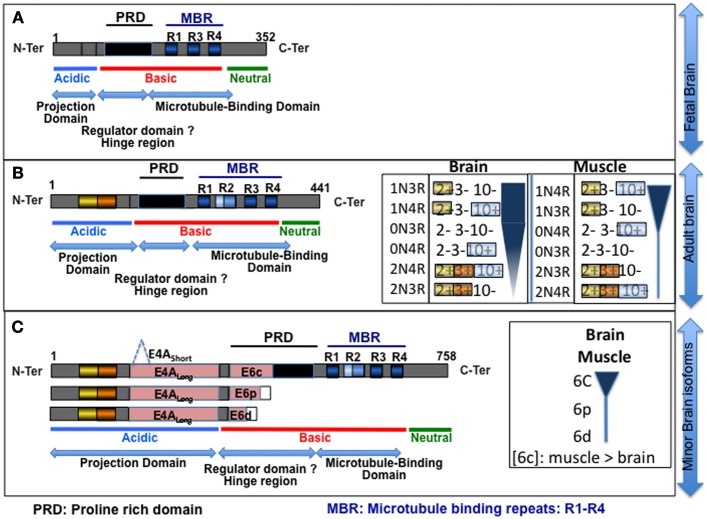
**Schematic of the Tau isoforms. (A,B)** isoforms primarily expressed in fetal and adult brains, respectively, **(C)** poorly expressed in brain. The different structural domains are indicated at the bottom of each panel. The inserts in **(B)** represent the differential expression of Tau isoforms in the adult brain, and the tissue specificity of the splicing is illustrated by comparison with skeletal muscle expression; the width of the diagram corresponds to level of the corresponding isoform [drawn from Mulot et al. (1994), Boutajangout et al. (2004), Andreadis (2005), Trabzuni et al. (2012)]. Exon 4A Short and Long and exons 6c, p, and d correspond to distinct 3′ sites in exons 4A and 6 (panel **C**) (Wei and Andreadis, 1998; Souter and Lee, 2010). The inclusion of 6p and 6d results in a change in the open reading frame (ORF) and introduces a stop codon, leading to the expression of truncated proteins. Note that the inclusion of exons 3, 6, and 4A occurs rarely in the brain. As shown in the insert in **(C)**, exon 6 is primarily detected in muscle compared with the brain (Andreadis, 2005). Exon 4A is primarily expressed in the peripheral nervous system (Couchie et al., 1992; Georgieff et al., 1993), and few data are available concerning this exon. PRD, proline-rich domain; MBR, microtubule-binding repeat: R1–R4.

The aggregate composition in Tau isoforms permits the classification of the different tauopathies into five sub-groups (reviewed in Sergeant et al., 2005). In the human adult brain, six Tau protein isoforms are expressed through alternative splicing of exons 2, 3, and 10 (Figures 2, 3). These isoforms are named according to their splicing patterns: 2N3R, 1N3R, 0N3R, 2N4R, 1N4R, and 0N4R. Two other minor exons, 4A and 6, have also been detected in mature brain RNA, but the levels of these transcripts are low, and the corresponding proteins are not detected in the brain. Depending on the disease, the Tau protein isoform composition varies. All six Tau isoforms are aggregated in some diseases, such as AD, whereas the preferential aggregation of 3R or 4R isoforms occurs in other diseases, such as Pick's disease (PiD) and progressive supranuclear palsy (PSP), respectively [reviewed in Sergeant et al. (2005)]. DM is the only disease characterized by the preferential aggregation of a single isoform: the smaller 0N3R isoform (Vermersch et al., 1996). The primary expression of the smaller protein could suggest mis-splicing of the Tau transcripts in DM.

Interestingly, NFTs have also recently been reported in sympathetic ganglions, suggesting a potential association with the peripheral neuropathy observed in some DM1 patients (Kuru et al., 2013; Peric et al., 2013b).

#### Other histological features

LBs, which are primarily composed of α-synuclein, are characteristic of DLB and Parkinson's disease (PD) but have also been observed in mixed AD/LB dementia (Rosenblum and Ghatak, 1979; Gibb et al., 1989; Bose et al., 2008; Echavarri et al., 2012). Interestingly, LBs have also been reported in DM brains in some patients (Itoh et al., 2010), suggesting a possible mixed pathology in these cases.

Analysis employing an anti-ubiquitin antibody revealed Marinesco bodies (MB) in the substantia nigra, which are rarely observed in DM1 patients but numerous in DM2 patients (Maurage et al., 2005). Although MBs have been detected in aging persons, the frequency of these inclusions increases in several diseases, such as DM.

Recently, a new histological feature has been reported in the hippocampal neurons of DM patients (Yamazaki et al., 2011; Nakamori et al., 2012). Granulovacuolar degeneration (GVD) is characterized by the presence of double membrane-bound cytoplasmic vacuoles, which are strongly detected using antibodies directed against late-stage autophagic marker Lamp1. These vacuoles contain an electron-dense granule that contains, at a minimum, endosomal sorting complex required for transport (ESCRT)-III subunits, charged multivesicular body protein 2B (CHMP2B), ubiquitin, pSmad2/3, and phospho-TDP-43 (Braak and Braak, 1991; Okamoto et al., 1991; Baig et al., 2009; Yamazaki et al., 2010; Funk et al., 2011). Although detected in the hippocampus in elderly persons, GVD is more frequently present in AD patients. The GVD load increases with disease severity, NFD, and the decline of episodic memory in AD (Ball, 1977; Ball and Lo, 1977; Ghoshal et al., 2002). GVD has also been reported in other neurodegenerative diseases, such as PiD, PSP, and cortico-basal degeneration (CBD) (Yamazaki et al., 2011).

## DM1 and Tau mis-splicing

### Evidence for Tau mis-splicing

Tau mis-splicing was first suggested by an abnormal pattern of pathological Tau proteins in the brain tissue of DM1 patients compared with the prototypical quadruplet of pathological tau proteins in the brains of AD patients (Vermersch et al., 1996). Tau mis-splicing in DM1 has clearly been demonstrated at the RNA and protein levels. Thus, in the DM1 brain, there is an overall reduction of Tau protein isoforms with the sequence encoded by exons 2 and 3 due to a deficit in the alternative splicing of these exons (Sergeant et al., 2001; Leroy et al., 2006a). Thus, an exon 2-specific antiserum reveals NFT staining in AD brains but not DM1 brains (Maurage et al., 2005). In addition to exons 2 and 3, the mis-splicing of Tau has also been reported for exon 10, although this inclusion defect occurs to a lesser extent compared with exon 2 and is not observed in all DM1 brains (Jiang et al., 2004; Dhaenens et al., 2011). We also reported the defective splicing of the minor Tau exon 6 brain cassette (Wei and Andreadis, 1998; Leroy et al., 2006a). Exon 6 is included in the mature mRNA through one of three potential 3′ splice sites, leading to the insertion of the 6c, 6p, or 6d forms (Figure 2; Andreadis, 2005). A decrease in exon 6c and an increase in 6d have been observed in DM1 brains (Leroy et al., 2006a). Although primarily expressed in the brain, Tau is also expressed in various tissues, and the alternative splicing of this protein is tissue specific. Interestingly, the alteration of Tau alternative splicing in DM1 also seems to be tissue specific, as mis-splicing of exon 2 is observed in both brain and skeletal muscle, whereas mis-splicing of exon 6 (decrease in exon 6c and increase in 6d) is only observed in the brain (Leroy et al., 2006a).

### Mechanisms

Two protein families, CELF and MBNL, have been implicated in the mis-regulation of the alternative splicing of muscle and heart transcripts in DM1, including cTNNT2, IR, and ClC-1 (Philips et al., 1998; Ladd et al., 2001; Savkur et al., 2001; Charlet-B et al., 2002; Kanadia et al., 2003; Ho et al., 2004; Kino et al., 2009). In these tissues, CELF1 and MBNL1 are antagonistic regulators of many splicing events altered in DM1, and these events are oppositely regulated during development: CELF1 expression decreases whereas MBNL1 increases during heart development in mice (Kalsotra et al., 2008). Thus, the sequestration of MBNL1 by mutated RNAs and the steady-state level of CELF1 in DM1 tissues reproduces the fetal level of these factors, leading to a fetal-type pattern of splicing [recently reviewed in Lee and Cooper (2009), Mastroyiannopoulos et al. (2010), Klein et al. (2011)].

Studies aiming to understand the pathogenic mechanisms involved in mis-splicing events in the brain are less numerous than those concerning mis-splicing in skeletal muscle and the heart. Only Tau is currently being studied. The eventual role of CELF and MBNL proteins in Tau mis-splicing has been investigated for exons 2, 6, and 10, as described below. No data are available concerning the dysregulation of Tau exon 3 splicing. This cassette is rarely included in adult brain Tau transcripts, and few data are available concerning the normal splicing regulation of this exon. However, studies have shown that the inclusion of exon 3 is dependent on the insertion of exon 2 and a weak branch point/poly Y region (Wei and Andreadis, 1998; Arikan et al., 2002; Andreadis, 2005; Trabzuni et al., 2012).

#### Celf family involvement

CELF1 (CUG-BP1) was the first CELF protein to be associated with mis-splicing events in the muscle and heart of DM1 patients [reviewed in Cho and Tapscott (2007)]. CELF1 is inefficient in the splicing of Tau exons 2, 6, and 10 (Leroy et al., 2006b; Dhaenens et al., 2011). However, CELF2 (ETR-3) acts as a silencer for both exons 2 and 10 but is inefficient in silencing exon 6 (Leroy et al., 2006a,b; Dhaenens et al., 2011). The role of CELF4 is more ambiguous because this protein inhibits exon 2 inclusion, favors the inclusion of exon 10, and, similar to CELF2, inefficiently modifies exon 6 splicing (Leroy et al., 2006a,b; Dhaenens et al., 2011). CELF5 and CELF6 are the only two CELF members that modify exon 6 splicing and reproduce its DM1 splicing pattern when over-expressed via cell transfection (Leroy et al., 2006a). Notably, neither of these proteins is expressed in muscle; whereas CELF6 is expressed in a subset of tissues, such as brain, kidney, and testis, CELF5 is principally expressed in the brain (Ladd et al., 2001, 2004). This expression pattern could explain why Tau exon 6 mis-splicing is brain specific and suggests that CELF 5 and 6 might play a key role in this specific mis-splicing (Leroy et al., 2006b). These results confirm the complexity and independent regulation of Tau exon splicing in terms of expression profile and regulation, consistent with Andreadis's results (2005). The different levels of splicing alterations for each exon in DM1 might reflect this complexity. Several members of the CELF family could contribute to the global mis-splicing pattern of Tau in the same tissue, but each protein might play a specific role for a given Tau exon. Furthermore, the inefficient regulation of Tau splicing by CELF1 suggests that the Tau exon 2 mis-splicing reported in muscle does not result from an increase in CELF1 activity, similar to other DM1-regulated transcripts, such as Bin-1 (Fugier et al., 2011). Rather, Tau exon 2 mis-splicing might result from the activity of other CELF members, such as CELF2 (Leroy et al., 2006b). An increase in CELF2 activity has been reported in an inducible heart-specific DM1 mouse model (Wang et al., 2007). Similar to the brain, CELF factors other than CELF1 and CELF2 may participate in Tau splicing regulation in muscle; this mechanism has been proposed for other deregulated transcripts in DM. Indeed, Kino et al. reported that CELF 3, 4, 5, and 6 but not CELF1 and CELF2 regulate ClC-1 splicing (Kino et al., 2009).

The different roles of the CELF factors for different Tau exons could explain, at least in part, why the relative exclusion of the main mis-spliced Tau exons in DM, exon 2 and exon 10, differs. Indeed, the exclusion of exon 2 is more important than that of exon 10, and an increase in exon 10 exclusion was not observed in all DM1 brains, in contrast to exon 2 (Jiang et al., 2004; Dhaenens et al., 2011). The CUG expansion length and Tau polymorphism do not play a role in exon 10 mis-splicing susceptibility (Dhaenens et al., 2011). By contrast, variations in exon 10 splicing might be associated with CELF protein expression in the brain. Indeed, CUG-BP1 and CELF2 protein expression varies greatly in DM1 brains: both increased and decreased expression has been observed compared with controls. By contrast, the expression of CELF4 was stable and similar to that of the control (Dhaenens et al., 2011). Interestingly, the brains with greater CELF1 and 2 levels also exhibit variations in exon 10 splicing. Although these observations are correlative, these results suggest the stabilization and activation of at least some of the CELF proteins in the brain, perhaps through a phosphorylation mechanism as described for CELF1 in muscles (Kuyumcu-Martinez et al., 2007, Dhaenes and Sergeant unpublished result].

Moreover, the differing actions of CELF factors on different Tau exons support our hypothesis that Tau exons are independently regulated and further suggest that the splicing misregulations observed during DM1 do not reflect a unique mechanism, consistent with the hypothesis of Jiang et al. (2004) concerning APP, Tau, and NMDAR mis-splicing.

#### MBNL family involvement

Interestingly, by favoring fetal-type splicing events, the DM1 mutation favors the expression of long fetal MBNL1 isoforms in DM1 brains, as observed in DM1 muscles (Lin et al., 2006; Dhaenens et al., 2008). Notably, this change is compatible with an increase in MBNL1 nuclear localization and then potentially in its depletion through sequestration in foci (Tran et al., 2011). Mimicking the MBNL1 sequestration in foci, the silencing of MBNL1 expression in cellular models results in the loss of Tau exon 2 insertion, similar to that observed in DM1 brain splicing (Dhaenens et al., 2008, 2011). Surprisingly, Tau splicing remains unchanged in the brains of MBNL1 KO mice, a model that reproduces some of the muscle abnormalities, the cataracts, and the mis-splicing of some RNA transcripts, such as TNNT2 and TNNT3, observed in DM1 (Kanadia et al., 2003; Suenaga et al., 2012). In contrast to the MBNL1 KO model, the preferential exclusion of Tau exons 2 and 3 is observed in mice lacking MBNL2, a new model that reproduces the brain but not muscle pathology of DM1 (Charizanis et al., 2012). Exon 10 splicing has not been reported in this MBNL2 KO model. Similar to exon 2, MBNL1 silencing also favors exon 6 exclusion in a manner similar to that observed in the DM1 brain, whereas exon 10 splicing is not modified (Leroy et al., 2006a; Dhaenens et al., 2011). This last result is consistent with the differential regulation of exon 2 and 10 splicing, as reported in CELF regulation. However, the results of a recent study concerning the impact of MBNL1 silencing efficiency on the number and severity of splicing alterations in a myoblast model suggest a need to re-examine the influence of MBNL1 activity on Tau exon 10 regulation in a more robust inducible system using stable cell lines (Jog et al., 2012).

### Effects of DM1 mis-splicing on Tau function

Splicing is a discrete cellular mechanism to modulate protein function. Thus, mis-splicing leads to modifications of protein activity. Most of the tauopathies for which an alteration of Tau alternative splicing has been reported are characterized by an alteration of exon 10 inclusion/exclusion. Thus, three isoforms are expressed according to the insertion or exclusion of exons 2 and 3 (Sergeant et al., 2008). DM is the only tauopathy characterized by the extensive alteration of Tau splicing involving exons 2, 3, 6, and 10, resulting in the primary expression of only one isoform. Although the role of each exon-coded sequence is not well-defined, we will discuss the potential consequences of DM mis-splicing on Tau function.

#### Tau protein structure

Tau is a “natively unfolded” protein (Schweers et al., 1994; Jeganathan et al., 2008), but short sequences (maximum of 10 residues) transiently adopt secondary structures, facilitating transient functions such as protein-protein interactions (Mukrasch et al., 2009). In solution, Tau adopts a “paperclip” structure resulting from interactions between the N and C termini of the Tau and microtubule-binding domains (MBDs). This structure is independent of the presence/absence of exons 2, 3, and 10 (Jeganathan et al., 2008).

The Tau protein has been divided into various regions based on chemical (basic, acidic, neutral), biochemical (proline-rich domain (PRD), hinge region, repeats), or functional features (projection domain, microtubule-binding region). As shown in Figure 3, the insertion of alternatively spliced exons extends the different domains in which these sequences are inserted: exons 2, 3, and 4A lengthen the acidic region and, consequently, the projection domain, whereas exons 6c and 10 length the basic region, PRD, and MBR. None of the additional alternative sequences disrupt the properties of the domain in which they are inserted.

#### Effects of mis-splicing on Tau binding to membranes and its secretion

The N terminus of Tau (amino acids 2–18) has been implicated in its binding to the plasma membrane. Although the exon 2-encoded sequence is located proximal to this site, there are no reports on the influence of this sequence and its phosphorylation on Tau binding to membranes. However, an effect of the exon 2-encoded sequence on the secretion of N-terminal fragments has been reported (Kim et al., 2010).

#### Effects of mis-splicing on Tau aggregation

The microtubule-binding repeats (MBRs) are essential for Tau fibrillization, whereas the flanking regions are inhibitory (Wille et al., 1992; Alonso et al., 2001). Two short peptides encoded by exons 11 (PHF6) and 10 (PHF6^*^) have been identified as major factors in the aggregation of Tau (Von Bergen et al., 2000, 2001; Li and Lee, 2006). The first motif, encoded by exon 11, is sufficient to promote aggregation, whereas the second motif, encoded by exon 10, facilitates aggregation. Thus, a loss of Tau isoforms with 4 MBRs would prevent the aggregation of this protein.

The role of the exon 2-encoded sequence in Tau aggregation has recently been described. The exon 2-encoded sequence promotes the fibrillar extension of Tau filaments but does not promote the nucleation of Tau aggregates (Zhong et al., 2012). By contrast, the inclusion of the Tau exon 3-encoded sequence diminishes the fibrillar extension, consistent with a potential protective effect of exon 3, as suggested by its higher inclusion in association with the H2 haplotype, a protective haplotype with respect to tauopathy development, compared with H1 haplotype (Trabzuni et al., 2012). However, Tau hyperphosphorylation might interfere with the aggregation of Tau isoforms. Indeed, in a cellular model, Tau pseudo-phosphorylation at sites common to the different Tau isoforms inhibits the aggregation of all 3R isoforms but has little effect on the aggregation of 2N4R (2+3+10+ isoform) and 1N4R (2+10+ isoform), and enhances the aggregation of 0N4R (10+ isoform) (Combs et al., 2011).

Altogether, these observations suggest that the decrease in the inclusion of exons 2, 3, and 10 in DM1 might be protective with respect to Tau aggregation in DM1. However, note that the toxicity of aggregated tau is yet a matter of debate. A possible role of these aggregates in neuroprotection as well as a toxicity of soluble forms of Tau have been reported and recently reviewed in Cowan and Mudher (2013).

#### Effects of mis-splicing on protein-protein interactions

Tau carries many ionic charges and contains a PRD, which enable it to potentially interact with many protein partners; however, these interactions are transient and difficult to detect.

Interaction studies have primarily been performed using the longest isoform of Tau (2N4R) first identified in the brain or some region of this isoform [for a review, see Mandelkow and Mandelkow (2012)]. Only interactions with sequences encoded by exons 2, 3, and 10 might be reduced or promoted in DM1 compared to control. As described below, while Tau protein interactions with microtubules are dependent on the insertion or omission of the sequence encoded by exon 10, these interactions might also depend on the phosphorylation state of the protein, particularly in the MBR and neighboring regions [reviewed in Sergeant et al. (2005)]. HSP70 binding to Tau affects microtubule polymerization and efficiently inhibits Tau aggregation (Dou et al., 2003; Sahara et al., 2007; Voss et al., 2012). These effects are isoform dependent. The anti-aggregative effect of HSP70 is higher for 3R isoforms than 4R isoforms. Thus, these results are also in agreement with the moderate development of the tauopathy in the DM1 brain.

Exon 6c is the most frequent form of exon 6 inclusion, but no data concerning its possible interactions with other partners are available. Exon 6c inclusion lengthens the PRD, and the encoded sequence is rich in putative sites of phosphorylation, features that modulate protein functions and interactions. In addition to the 85 putative sites present on the 2N4R isoform, the exon 6c-encoded insert (66 amino acids) introduces 22 new putative sites of phosphorylation (21 Ser/Thr and 1 Tyr, i.e., 1/3 residues), four of which could be proline-dependent. However, the impact of the exon 6c-encoded sequence on Tau phosphorylation is yet unknown. Although Tau containing exon 6c-encoded sequence binds microtubules, it might serve as an inhibitor of axon elongation, particularly when the Tau isoforms also contain exon 2- and exon 3-encoded sequences (Luo et al., 2004a,b).

#### Effects of mis-splicing on microtubule stabilization and axonal transport

The MBR comprises three (3R) or four (4R) tubulin-binding repeats depending on the insertion or omission of the sequence encoded by exon 10. The insertion of a fourth microtubule-binding domain (the second one in the primary structure of the protein) enhances microtubule binding by Tau by 40-fold and, consequently, also enhances the stability of the microtubules (Goode and Feinstein, 1994). Consequently, 4R-Tau is a potent inhibitor of MT shortening, in contrast to the 3R-Tau isoforms (Bunker et al., 2004). In the human adult brain, the ratio between 3R and 4R is 1:1, and both the 3R and 4R proteins are detected throughout the brain (Mulot et al., 1994; Boutajangout et al., 2004; Trabzuni et al., 2012). Thus, the loss of the Tau 4R protein observed in DM1 might result in a loss of microtubule stabilization and an increase in neuronal plasticity.

The Tau 3R/4R balance could also affect other Tau properties. Indeed, Tau affects organelle transport by reducing the attachment frequency of motors to microtubules (Sato-Harada et al., 1996; Trinczek et al., 1999; Seitz et al., 2002; Mandelkow et al., 2004) or differentially regulating kinesin- and dynein-based transport (Dixit et al., 2008; Vershinin et al., 2008). According to Dixit et al. (2008), Tau interferes with anterograde transport by inducing kinesin detachment. However, by reversing the direction of dynactin-dynein motor proteins, Tau also interferes, to a lesser extent, with retrograde transport. The perturbations of kinesin and dynein-dynactin migration are both dependent on the Tau isoform; the shortest Tau isoform (0N3R) is a more potent inhibitor than the longest isoform (2N4R) (Dixit et al., 2008). Thus, the overexpression of the fetal form (0N3R) and the disappearance of the adult forms might lead to an increased perturbation of axonal transport in the DM1 brain compared to other tauopathies, such as AD, as well as increased neuronal plasticity. Interestingly, both the N- and C-halves of Tau interfere with kinesin-mediated axonal transport. First, Tau 3R has a more significant effect than Tau 4R on both kinesin and dynein binding and progression along stabilized microtubules (Vershinin et al., 2007, 2008; Stoothoff et al., 2009), while Tau 4R but not Tau 3R enhances the velocity of kinesin (McVicker et al., 2011). Second, the N terminus inhibits fast anterograde transport (FAT) by activating the PP1–GSK3 pathway due to the exposure of the phosphatase-activating domain (PAD) in aggregated Tau (LaPointe et al., 2009; Kanaan et al., 2011). However, the phosphorylation of Tau Y18, an AD-specific phosphorylation site and Fyn substrate, prevents the effect of Tau on FAT (Kanaan et al., 2012). Note that sequences encoded by exons 2 and 3 lengthen the Tau projection domain, resulting in an increase in the space between microtubules (Kanai et al., 1992). Thus, these sequences might reinforce the effects of Tyr18 phosphorylation on axonal transport. Indeed, the space between microtubules was recently shown to be a potential determinant in mitochondrial transport (Shahpasand et al., 2012).

Axonal transport also depends on Tau exon 6 splicing. The 6p- and 6d-containing forms are sufficient to perturb FAT because these short isoforms cannot adopt the “paperclip conformation” in the absence of the C-terminus, leading to the spontaneous exposure of the PAD domain (LaPointe et al., 2009; Kanaan et al., 2011). Thus, although exon 6 is a minor cassette in DM1, the increase in 6d levels could contribute, even weakly, to the disruption of axonal transport in DM1 patients.

In conclusion, by favoring the fetal Tau isoform (0N3R) and increasing the minor 6d forms, changes in Tau alternative splicing might result in a variation of microtubule bundle organization and axonal transport in the DM1 brain and an enhanced secretion and diminished aggregation of Tau.

## Common features of DM and other tauopathies

The term tauopathy merges nearly 30 diseases characterized by Tau aggregation and neurodegeneration due to various factors, such as genetic (mutations or polymorphisms/haplotypes of different genes), environmental (trauma), and molecular factors (with or without amyloid cascade contributions) [reviewed in Sergeant et al. (2005)]. In DM, the expansion of oligonucleotide repeats (CTG/CCTG) leads to tauopathy. Thus, there are interconnections of the different pathways that are initiated by distinct factors and lead to both tau aggregation and neurodegeneration. What are the common determinants and crucial steps of these different diseases that contribute to tauopathy development?

### Tau mis-splicing

Tau mis–splicing has been implicated in tauopathy development since the discovery that Tau mutations in intronic sequences resulted in both splicing deregulation and tauopathy in FTDP-17 cases. Indeed, Tau mis-splicing has been identified in various tauopathies, such as FTDP-17, PSP, CBD, argyrophilic grain disease, DM, and, to a lesser extent, AD and Down syndrome (Sergeant et al., 2005). Notably, the mechanisms of mis-splicing might differ according to the disease. In FTDP-17, mis-splicing is due to *cis*-factors, i.e., Tau mutations, at sites involved in splicing factor binding. In DM, mis-splicing is due to a disruption in the ratio of some splicing regulator *trans*-factors, particularly those belonging to the CELF and MBNL families. In other cases, such as PSP and CBD, the mechanisms differ according to familial or sporadic forms of the disease. In familial cases, Tau 4R over-expression results from Tau mutations in the binding site of regulator factors. In sporadic forms, Tau 4R over-expression is dependent on the Tau haplotype. Indeed, the H1 *MAPT* haplotype has been consistently associated with PSP (Rademakers et al., 2005). Down-regulation of miR-132 in the brains of PSP patients was recently reported. Furthermore, silencing of this miR leads to an increase in the ratio of the Tau 4R/3R isoforms (Smith et al., 2011). Thus, in some tauopathies, mis-splicing could be associated with variations in miR expression.

Taken together, these data indicate that dysregulation of the balance between the different Tau isoforms is a sufficient factor to trigger tauopathy. Although an increase in Tau 4R levels is the most frequent dysregulation observed in FTDP-17, PSP, and CBD, the overexpression of 3R isoforms is associated with tauopathies such as rare FTDP-17 cases and PiD. In these different pathologies, no splicing error in exons 2 or 3 has been reported. The DMs are the first pathologies in which global indirect Tau mis-splicing, i.e., errors in the splicing of exons 2, 3, 6, and 10, have been observed (Sergeant et al., 2001, 2008; Jiang et al., 2004; Leroy et al., 2006b).

Conflicting data concerning AD have been reported. Mis-splicing is difficult to demonstrate in whole tissues because only some neurons, particularly cholinergic neurons, are degenerating. The number of degenerating neurons is a function of the area and stage of the pathology. Thus, some studies have not detected changes in splicing during AD, while others have reported an increase in exon 10 inclusion, although this increase is lower than that observed for other tauopathies (Baker et al., 2000; Boutajangout et al., 2004; Connell et al., 2005; Glatz et al., 2006; Ingelsson et al., 2006; Conrad et al., 2007; Abraham et al., 2009). Only one study reported an alteration in exon 2 splicing, which involved a decrease in exon 2 inclusion. Interestingly, an increase in exon 3 inclusion has been reported in cells and subjects with the H2 haplotype (Conrad et al., 2007; Caffrey et al., 2008; Wegiel et al., 2011; Trabzuni et al., 2012), which is protective compared with H1. A study of Down syndrome patients developing AD with aging suggests an increase in exon 10 exclusion (Wegiel et al., 2011).

### Diabetes and metabolic syndrome

Another common feature of DM1 and some tauopathies involves insulin metabolism and associated diseases. Although the importance of insulin resistance in DM remains controversial (Perseghin et al., 2004), DM1 patients exhibit peripheral insulin resistance with glucose intolerance, hyperinsulinemia, and an increased risk of developing type II diabetes (T2DM) (5–17% of patients) (Moxley et al., 1984; Savkur et al., 2001; Rakocevic Stojanovic et al., 2010; Kaminsky et al., 2011). This peripheral insulin resistance might be due to the IR splicing alterations observed during DM (Savkur et al., 2001, 2004). However, the relationship between IR splicing, insulin resistance, and T2DM is unclear. Although the overexpression of IR A (i.e., the IR isoform without the exon 11-encoded sequence) has been reported in one T2DM case with extreme insulin resistance and hyperinsulinemia (Norgren et al., 1994), a decrease in the IR A form has been more frequently reported in T2DM (Mosthaf et al., 1991; Sesti et al., 1991; Kellerer et al., 1993; Norgren et al., 1993). However, this alteration has not been observed by other groups (Benecke et al., 1992; Anderson et al., 1993; Hansen et al., 1993). By contrast, an increase in IR A expression similar to that observed in DM1 has been evidenced in obese and early-stage diabetic rhesus monkeys, supporting the hypothesis of a relationship between IR splicing and diabetes (Huang et al., 1994, 1996).

Interestingly, metabolic syndromes, including T2DM, are also a significant risk factor for AD [reviewed in Frisardi et al. (2010), Bosco et al. (2011)]. Hyperglycemia leads to an excessive peripheral utilization of insulin, resulting in reduced insulin transport to the brain. Brain insulin plays a role in neuron survival, learning memory, synaptic plasticity, and neuronal energy homeostasis [reviewed in Belfiore et al. (2009)]. Intranasal administration of insulin has potential beneficial cognitive effects, confirming the possible deleterious impact of insulin in AD (Reger et al., 2008; Shemesh et al., 2012). Insulin could interfere with pathological processes at various stages. First, insulin resistance has been suggested to be involved in cognitive impairment (Bruehl et al., 2010). Second, insulin might accelerate AD-related pathology through its effects on amyloid beta (Aβ) metabolism and Tau phosphorylation [reviewed in Bosco et al. (2011)]. Interestingly, intranasal insulin partially corrects Tau hyperphosphorylation in diabetic rats (Yang et al., 2013). Third, the increased expression of three-repeat isoforms of Tau could contribute to the Tau pathology in a rat model of chronic type 2 diabetes (Jung et al., 2011). Notably, increases in both Tau phosphorylation and Tau 3R levels have also been observed in DM1 patients. Furthermore, in the absence of peripheral insulin resistance but up-regulation of hippocampal insulin signaling, early and progressive obesity potentiated spatial learning deficits and hippocampal Tau pathology at a later stage in transgenic THY-Tau22 mice (Leboucher et al., 2013). Interestingly, Tau phosphorylation and/or Aβ oligomerization/aggregation have been observed in several diabetic mouse or rabbit models, confirming a possible relationship between AD disease and diabetes (Li et al., 2007; Planel et al., 2007; Jolivalt et al., 2010; Bitel et al., 2012; Papon et al., 2013).

### Common molecular actors in DM and AD

Various molecular actors are common between DM and tauopathies such as AD and are therefore of particular interest for understanding pathological processes. The contribution of these different actors to Tau aggregation and neurodegeneration has not been well-elucidated. Nevertheless, the deregulation of the function of these proteins could be a metabolic event contributing to the common pathological aspects of these diseases, i.e., the development of a tauopathy. Thus, we will discuss these common actors and how they are involved in either DM or tauopathy.

#### Bin-1

Tau is not the only transcript targeted by the DM1 mutation that is ubiquitously expressed, but the DM1 mis-splicing is often reported for only one tissue. For Bin-1, the preferential exclusion of muscle-specific exon 11 has been reported in DM1 muscle compared with healthy persons and has been associated with T tubule alterations and muscle weakness (Fugier et al., 2011). Other exons of Bin-1 such as exons 7 and 13–16, are brain specific, but their eventual mis-splicing has not been investigated, and therefore the contributions of these entities cannot be ruled-out. Although Bin1 mis-splicing has not been established in the DM brain, Bin1 is a recently identified genetic marker for predisposition to the most frequent tauopathy, AD (Seshadri et al., 2010; Carrasquillo et al., 2011; Lambert et al., 2011; Logue et al., 2011; Wijsman et al., 2011). A particular variant, located 28 kb upstream of Bin-1, has been associated with risk for AD, particularly the Tau load in the brains of AD patients. This SNP results in the increased expression of Bin-1. Bin-1 is highly expressed in neuronal tissue, and its role in neurons must be accurately determined. Bin-1 is suspected to interfere with synaptic function and cell membrane processes and might be associated with receptor-dependent signaling pathways (Leprince et al., 1997; Morgan, 2011). Interestingly, Bin-1 interacts with Tau proteins (Chapuis et al., 2013).

#### APP

Jiang et al. (2004) reported a default in APP splicing in DM1 patients, characterized by a decrease in exon 7 inclusion. The sequences encoding exons 7 and 8 are specifically excluded from the neuronal APP form (APP695) but are included in astrocytic APP isoforms (APP 751, APP770). Interestingly, a change in the APP splicing pattern has also been observed in the brains of AD patients. In contrast to the DM brain, the modified splicing corresponds to an increase in exon 7 inclusion in the AD brain (Johnson et al., 1989; Tanaka et al., 1989). However, changes in APP splicing are somewhat difficult to interpret because APP is expressed in both neurons and astrocytes. An increase in exons 7 and 8 might either correspond to the mis-splicing of APP transcripts in neurons or result from neuronal loss or astrocytosis, both events observed in AD (Donev et al., 2009; Rodriguez et al., 2009). In DM1, a decrease in exon 7 inclusion might be indicative of mis-splicing in astrocytes. The functional consequences of changes in APP splicing in astrocytes remain ill-defined.

In addition to mis-splicing, defects in APP metabolism have been strongly associated with the development of some tauopathies, particularly AD [recently reviewed in Huang and Mucke (2012)]. Indeed, APP proteolysis is modified during AD, favoring the production of Aβ 1-42 or Aβ N-42 peptides, which aggregate in the extracellular space. Aβ 1-42 dosage is one of the three biological markers indicative of an AD diagnosis: Aβ 1-42 is decreased in the cerebrospinal fluid (CSF) of AD patients, whereas total Tau and phosphorylated Tau levels are increased (Blennow and Hampel, 2003; Herukka et al., 2005; Bombois et al., 2013). Decreased Aβ 1-42 levels in the CSF have also been observed in various tauopathies and other diseases, such as Creutzfeld-Jacob disease and Multiple System Atrophy (Van Everbroeck et al., 1999; Otto et al., 2000; Sjogren et al., 2002; Noguchi et al., 2005). Interestingly, the analysis of CSF in DM patients also revealed a significant decrease in Aβ 1-42 compared with matched controls (Winblad et al., 2008; Peric et al., 2013a). While a decrease in the Aβ 1-42 level in the CSF of AD patients is associated with the presence of the cerebral aggregation of this peptide, this is not the case for other tauopathies, particularly DM (Kiuchi et al., 1991; Figure 1).

#### NMDAR

In the DM1 brain, defective splicing of NMDA-R1 is observed, which, in conjunction with altered Tau splicing, suggests defective neuronal plasticity. This splicing change corresponds to an increase in exon 5 inclusion in NMDAR1 transcripts (Jiang et al., 2004) and could result in the functional modification of the protein, although this possibility remains to be determined. N-methyl-D-aspartate receptors (NMDAR) are multimeric ligand-gated ion channels comprising at least one member of the NMDA receptor 1 subunit (NMDAR1) family and one of four different subunits of the NR2 family (NMDAR2 A-D). These receptors play a role in neuronal plasticity, but over-activation of these receptors results in excitotoxic damage (Chandler et al., 1998; Jaekel et al., 2006). The NMDAR1 family comprises eight alternatively spliced variants generated through the alternative splicing of exons 5, 21, and 22 (Laurie and Seeburg, 1994; Zukin and Bennett, 1995). The splicing pattern varies during development, across brain regions, and among subtypes of neurons. The presence of exon 5-encoded sequences determines the sensitivity of NMDAR to protons and polyamines and accelerates receptor deactivation after brief agonist exposure (Traynelis et al., 1995, 1998; Rumbaugh et al., 2000).

Interestingly, a pivotal role for NMDAR-mediated toxicity has been suggested in AD and tauopathies. Indeed, an overactivation of N-methyl-D-aspartate glutamate (NMDA) receptors, which permits excessive Ca^2+^ influx through associated ion channels, would induce damage and neuronal cell death. NMDAR function can also be modulated through the metabolite products of APP and Tau, proteins essential to AD development. Indeed, the over-expression of human Tau and its N-terminal fragments in primary neuronal cultures leads to N-methyl-D-aspartate receptor (NMDAR)-mediated cell death. As a feedback loop, NMDAR stimulation causes calpain activation, followed by Tau protein proteolysis, resulting in highly toxic N-terminal peptides, such as the 17-kDa peptide (Amadoro et al., 2006). This feedback might be associated, at least in part, with the presence of Tau in the dendritic spines and the axonal localization of this protein. Moreover, naturally secreted Aβ dimers and trimers but not monomers induce the progressive loss of hippocampal synapses, mediated through the activity of NMDARs (Shankar et al., 2007). Thus, NMDAR antagonists, such as Memantine, a drug that blocks ion channel formation mediated through NMDARs, are presently used in AD treatment [reviewed in Schmitt (2005) and Dominguez et al. (2011)]. Memantine might also inhibit Tau and APP protein translation, mediated through the internal ribosome entry site (IRES) of their transcripts. Thus, Memantine inhibits the neuronal expression of Tau and APP proteins, two essential actors in AD (Wu and Chen, 2009). Memantine has also been reported to block the increase in Ca^2+^ flux and oxidative stress induced through soluble Aβ oligomers (De Felice et al., 2007).

#### CELF2

CELF proteins, particularly CELF1 and CELF2, play an important role in DM1 mis-splicing. In the brain, CELF2 regulates the splicing of NMDAR exon 5 and Tau exons 2 and 3, thus acting as a silencer, whereas CELF1 is inefficient (Zhang et al., 2002; Leroy et al., 2006a; Dhaenens et al., 2011). Curiously, the genome-wide association of familial late-onset AD with CUGBP2 SNPs has been reported in patients with ApoE e4/e4 genotypes, i.e., patients with high risk of developing late-onset AD (Wijsman et al., 2011). However, the relationship between CELF2 SNPs and AD has not been elucidated.

## Beyond tauopathy

### A toxic gain-of-function of RNA

As detailed in the two preceding sections, Tau mis-splicing induced by CTG mutation has been suggested to be sufficient to induce tauopathy. But is tauopathy sufficient to explain all the brain defects in DM1? Animal models could be useful to answer this question. Two animal models have been described to develop brain pathology: the MBNL2-KO mouse model and transgenic CUG SXXL mice (Gomes-Pereira et al., 2007; Charizanis et al., 2012; Huguet et al., 2012). To date, no data concerning an eventual tauopathy in these models have been reported.

Interestingly, Tau pathology has also been observed in some mixed *amyotrophic lateral sclerosis* (ALS)/FTD cases bearing GGGGCC expansions in C9ORF72, suggesting that some patients with nucleotide repeat expansions could develop a mixed pathology (Bieniek et al., 2013; King et al., 2013). Moreover, this observation also suggests a close relationship between oligonucleotide expansion, neuropathology, and tauopathy, and in the absence of mis-splicing, tauopathy could be a secondary consequence of neuropathy associated with oligonucleotide repeat expansion. Thus, it would be interesting to identify tauopathy in other diseases with repeat expansions.

However, other observations argue that some neurological dysfunctions occur independent of tauopathy development. Indeed, it is strange that all expanded repeat mutations, which cause more than 20 diseases, lead to neuropathologies. Why is this type of mutation preferentially associated with this type of disease? The repeat expansions differ with respect to their identity, number, and functional consequences and are harbored by different genes, leading to differential patterns of tissue expression. These pathologies present divergent pathological pathways; in particular, these expansions can either affect protein function or result in a gain of toxic function of the mutated RNA. A gain of toxic function of the mutated RNA was first reported in a study concerning DM1 and subsequently reported for other pathologies, such as HDL2, SCA8, 10, 31, FXTAS, and more recently, FTD, *amyotrophic lateral sclerosis* (ALS), mixed ALS/FTD cases, and SCA36 [reviewed in La Spada and Taylor (2010), DeJesus-Hernandez et al. (2011), Renton et al. (2011), Wojciechowska and Krzyzosiak (2011), Garcia-Murias et al. (2012), Ikeda et al. (2012), Simon-Sanchez et al. (2012)]. These pathologies are characterized by a nuclear aggregation of mutated transcripts (called foci) and a change in the alternative splicing of numerous transcripts. The protein trapped by the foci is dependent on the pathologies. Indeed, nuclear foci can sequester MBNL1 proteins in pathologies with CUG, CCUG, or CAG, CGG repeat expansions (Miller et al., 2000; Fardaei et al., 2002; Rudnicki et al., 2007; de Mezer et al., 2011) and hnRNPK in SCA10 (White et al., 2010) and Sam68 in FXTAS (CGG expansion), which also recruit MBNL1 and hnRNPG in foci (Iwahashi et al., 2006; Sellier et al., 2010). Recent reports have suggested that the distinction between diseases with expanded oligonucleotide repeats characterized by a gain of RNA function and those characterized by a gain of protein function is not so obvious [reviewed in Batra et al. (2010)]. SCA8 is the first disease for which bidirectional transcription leading to both RNA and protein dysregulation has been reported (Moseley et al., 2006). Indeed, a newly defined molecular mechanism, “repeat-associated non-ATG initiated translation (RAN translation),” results in the accumulation of SCA8 polyalanine and DM1 polyglutamine in, respectively, SCA8 and DM1 human tissues and mouse models (Zu et al., 2011). Nevertheless, the contribution of these proteins to disease pathology has not been elucidated. Similarly, both nuclear foci and polyglutamine protein aggregation have been reported in HDL2, another CTG expansion disease (Holmes et al., 2001; Rudnicki et al., 2007; Wilburn et al., 2011). Other mechanisms, such as haploinsufficiency, anti-sense RNA, and miRNA, have been suggested to interfere with pathology development [recently reviewed in Klein et al. (2011), Sicot et al. (2011); Udd and Krahe (2012)]. Thus, the neurological specificity of these pathologies might be determined by the as-yet undetermined pathological processes that they share.

### Spliceopathy and neurological disorders

Alternative splicing is involved in numerous neurological diseases and can be associated with either the disruption of *cis*-splicing sites or *trans*-acting factors [reviewed in Mills and Janitz (2012); Feng and Xie (2013)]. In addition to diseases with a gain of toxic RNA function (described above) and tauopathy associated with Tau mis-splicing (see Part III), a relationship between splicing and neuronal dysfunction has been demonstrated for other neurological pathologies, such as spinal muscular atrophy (SMA). SMA is a recessive autosomal disease associated with the dysfunction of the survival of motor neurons protein (SMN), a RNA-binding protein required for the efficient assembly of small nuclear ribonucleoprotein (snRNP) complexes and spliceosomal snRNP biogenesis. This protein has therefore been indirectly implicated in general cellular RNA processing. By modifying the pre-mRNA splicing machinery, mutated SMN might interfere with mRNA biogenesis (Lefebvre et al., 1995; Pellizzoni et al., 2002; Zhang et al., 2008). Moreover, Lotti et al. (Lotti et al., 2012) recently demonstrated that SMN interferes with the U12 splicing mechanism, a minor alternative process to classical splicing, and SMN depletion alters the splicing of 18 exons, particularly in *Stamsimon* transcripts, resulting in motor neuron degeneration. SMN also binds FMRP, a defective protein in Fragile X mental retardation syndrome that is associated with mRNA transport and translation (Piazzon et al., 2008).

Other neurological pathologies associated with the dysfunction of RNA-binding factors include ALS and FTD, which is induced by mutations in the *TDP-43* and *FUS* genes (Kabashi et al., 2008; Sreedharan et al., 2008; Vance et al., 2009). TDP43 regulates the splicing of several human transcripts, particularly those of SMN, serine/arginine-rich splicing factor 2 (SC35), S6 kinase 1(S6K1), and Aly/REF-like target (SKAR), a component of the exon junction complex (EJC) (Bose et al., 2008; Dreumont et al., 2010; Tollervey et al., 2011; Fiesel et al., 2012). Consistent with its function as an RNA-binding protein, TDP-43 associates with many other RNA-binding proteins, such as hnRNP A2/B1, hnRNP A1, hnRNP C1/C2, hnRNP A3, and FUS (which is involved in splicing processes and whose mutations are responsible for 5% of FTD) (Yang et al., 1998; Buratti et al., 2005; Freibaum et al., 2010; Ling et al., 2010). Furthermore, the loss of TDP-43 has recently been associated with long pre-mRNA depletion, RNA mis-splicing (Polymenidou et al., 2011; Tollervey et al., 2011), and a potential role for TDP-43 in axonal mRNA regulation and miRNA biogenesis associated with neuronal outgrowth (Fallini et al., 2012; Kawahara and Mieda-Sato, 2012). The role of FUS, the second protein involved in ALS/FTD, as a brain RNA splicing regulator (Rogelj et al., 2012), particularly for Tau and RNA-binding protein splicing (Orozco et al., 2012; Nakaya et al., 2013), has only recently been described. Both FUS and TDP-43 preferentially bind to extra-long introns, likely to protect these regions from incorrect splicing at cryptic sites (Lagier-Tourenne et al., 2012).

Although mis-splicing has clearly been demonstrated for various pathologies, including some tauopathies, recent studies have indicated the dysfunction of different splicing regulators in other diseases; however, a clear relationship with a particular splicing event has not been established in these latter diseases [see review Mills and Janitz (2012)]. The hnRNP A1 polymorphism is a genetic factor for some FTLD (Villa et al., 2011), and mutated RBFox1 has been associated with mental retardation, epilepsy, and autism spectrum disorders (Bhalla et al., 2004; Barnby et al., 2005; Martin et al., 2007; Sebat et al., 2007). SRP20, TDP-43, hnRNP-A/B, and CELF2 have been associated with AD (Amador-Ortiz et al., 2007; Uryu et al., 2008; Wijsman et al., 2011; Berson et al., 2012; Wong et al., 2012), and SFPQ has been associated with AD and PiD (Ke et al., 2012). These observations suggest that the involvement of RNA-binding proteins, particularly splicing regulator factors, largely contributes to the neurodegenerative process. Indeed, these factors can be involved in numerous splicing events, and the modulation of their activity resulting from the modification of the expression and localization of these factors could explain the variations in the alternative splicing of various transcripts detected in neurodegenerative diseases such as AD, Parkinson's disease, and schizophrenia (Twine et al., 2011; Cohen et al., 2012; Soreq et al., 2012; Wu et al., 2012; Wong, 2013) [recently reviewed in Cooper-Knock et al. (2012)]. However, these factors assume other functions, such as RNA export and RNA stabilization. Thus, a role of these factors in pathology through mechanisms other than splicing alterations cannot be excluded.

The deregulation of splicing could also result from snRNP deregulation, as recently suggested for AD. The accumulation of both U1SnRNP and U1-70K, elements essential to the splicing mechanism, has recently been associated with AD and unspliced RNA accumulation (Bai et al., 2013).

Taken together, these examples indicate that the modification of the splicing factor pool and the deregulation of snRNPs involved in splicing exert a toxic effect in humans; thus, the modification of the splicing pool induced by DM1 mutations might also be associated with neuronal dysfunctions other than tauopathies and Tau splicing alterations.

## Conclusion

**Figure 4:**
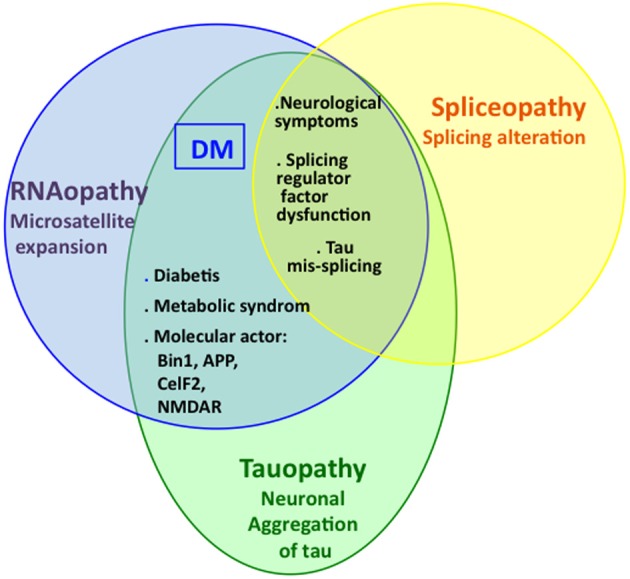
**The complex interaction between RNAopathy, spliceopathy, and tauopathy**. DM diseases are the first pathologies for which these interactions have been established.

DM is the first pathology identified as a combination of tauopathy, spliceopathy, and RNAopathy. Indeed, DM1 is the first identified pathology for which the tauopathy has been associated with an expansion of oligonucleotide repeats (Sergeant et al., 2001). It has been hypothesized that this DM-associated tauopathy might result from Tau mis-splicing induced through RNAopathy. This relationship between Tau mis-splicing and tauopathy has previously been reported for some FTD, and particularly for some cases of FTDP-17 with specific Tau mutations. However, Tau mis-splicing is only one among other molecular mechanisms for the induction of tauopathy. Indeed, other tauopathies have been associated with mutations in different genes: *APP, PS1*, and *PS2* (three genes involved in the amyloid cascade in AD); *MAPT* (Tau gene) (other mutations than those involved in splicing default), or the *MAPT* haplotype. Thus, various pathological mechanisms lead to tauopathy development, such as Tau mis-splicing in some cases of FTDP-17 and the amyloid cascade in AD, explaining why these pathologies, although clinically different, share several molecular features, as described in this review and schematized in Figure 4.

A complex interaction between RNAopathy, spliceopathy, and proteinopathy other than tauopathy has also been observed in other neurological diseases (Figure 4). Although these pathologies differ according to the nature and length of the microsatellite repeats, the aggregated proteins, clinical symptoms, and histological features, this interaction between RNAopathy, spliceopathy, and proteinopathy might be an essential inducer of the pathological mechanisms specific for neurological deregulation. Given the association between tauopathy, spliceopathy and RNAopathy in DM, it would be interesting to determine whether tauopathies are also associated with other diseases involving microsatellite expansions and investigate whether this association is not specific to DM but is rather a more global mechanism in which spliceopathy, RNAopathy, and proteinopathy converge.

### Conflict of interest statement

The authors declare that the research was conducted in the absence of any commercial or financial relationships that could be construed as a potential conflict of interest.

## Abbreviations

3′ UTR: 3′ untranslated region
Aβ: amyloid beta
AD: Alzheimer's disease
ALS: amyotrophic lateral sclerosis
APP: amyloid-β precursor protein
BBB: blood-brain barrier
Bin-1: bridging integrator-1
CBD: corticobasal degeneration
CELF: CUG-BP and ETR-3-like factor
CELF1: CUG-binding protein 1 (CUG-BP1)
CELF2: CUGBP2 or ETR-3
CNS: central nervous system
CSF: cerebrospinal fluid
DLB: dementia with Lewy bodies
DM: myotonic dystrophy
DM1: myotonic dystrophy of type 1
DM2: myotonic dystrophy of type 2
DMPK: myotonic dystrophy protein kinase
DS: Down syndrome
EDS: excessive daytime sleepiness
FAT: fast anterograde transport
FISH: fluorescence *in situ* hybridization
FTD: frontotemporal dementia
FTDP-17: frontotemporal dementia and parkinsonism linked to chromosome 17
FUS: fused in sarcoma
GVD: granulovacuolar degeneration
*hnRNP*: heterogeneous nuclear ribonucleoprotein
IR: insulin resistance
IRES: internal ribosome entry site
KO: Knockout
LB: Lewy bodies
MAPT: microtubule-associated protein tau
MB: Marinesco bodies
MBNL: Muscleblind-like
MBR: microtubule-binding repeats
MRI: magnetic resonance imaging
NFD: neurofibrillary degeneration
NFT: neurofibrillary tangle
NMDAR: N-methyl-D-aspartate receptors
ORF: open reading frame
PAD: phosphatase activating domain
PD: Parkinson's disease
PiD: Pick's disease
PRD: proline-rich domain
PROMM: proximal myotonic myopathy
PS: presenilin
PSP: progressive supranuclear palsy
RAN: Repeat-associated non-ATG-initited translation
RBP: RNA-binding proteins
SMA: spinal muscular atrophy
SMN: survival of motor neurons
SNPs: single nucleotide polymorphisms
snRNP: small nuclear ribonucleoprotein
T2DM: type II diabetes
TDP-43: TAR DNA-binding protein 43.
